# Exploiting Focused Ultrasound to Aid Intranasal Drug Delivery for Brain Therapy

**DOI:** 10.3389/fphar.2022.786475

**Published:** 2022-04-14

**Authors:** Gaetano Barbato, Robert Nisticò, Viviana Triaca

**Affiliations:** ^1^ Inno-Sol Srl, Rome, Italy; ^2^ Department of Biology, School of Pharmacy, University of Tor Vergata, Rome, Italy; ^3^ Laboratory of Pharmacology of Synaptic Plasticity, Fondazione EBRI Rita Levi Montalcini, Rome, Italy; ^4^ Institute of Biochemistry and Cell Biology (IBBC), National Research Council (CNR), International Campus A. Buzzati-Traverso, Rome, Italy

**Keywords:** neurodegenerative diseases, brain circuit vulnerability, drug delivery, focused ultrasound, clinical trials

## Abstract

Novel effective therapeutic strategies are needed to treat brain neurodegenerative diseases and to improve the quality of life of patients affected by Alzheimer’s disease (AD), Parkinson’s disease (PD), Huntington’s disease (HD), Amyotrophic Lateral sclerosis (ALS) as well as other brain conditions. At present no effective treatment options are available; current therapeutics for neurodegenerative diseases (NDs) improve cognitive symptoms only transiently and in a minor number of patients. Further, most of the amyloid-based phase III clinical trials recently failed in AD, in spite of promising preclinical and phase I-II clinical trials, further pinpointing the need for a better knowledge of the early mechanisms of disease as well as of more effective routes of drug administration. In fact, beyond common pathological events and molecular substrates, each of these diseases preferentially affect defined subpopulations of neurons in specific neuronal circuits (selective neuronal vulnerability), leading to the typical age-related clinical profile. In this perspective, key to successful drug discovery is a robust and reproducible biological validation of potential new molecular targets together with a concomitant set up of protocols/tools for efficient and targeted brain delivery to a specific area of interest. Here we propose and discuss Focused UltraSound aided drug administration as a specific and novel technical approach to achieve optimal concentration of the drug at the target area of interest. We will focus on drug delivery to the brain through the nasal route coupled to FUS as a promising approach to achieve neuroprotection and rescue of cognitive decline in several NDs.

## Introduction

Alzheimer’s disease (AD) and other neurodegenerative diseases (NDs) affect over 50 million people worldwide according to World Health Organization (WHO), with nearly 10 million new cases per year, projected to reach 82 million in 2030 and 152 in 2050 because of a rise in life expectancy to 60 years aged population (World Health Organization, 2017). A number of initiatives to prioritise dementia in the European policy agenda have been established by EU (https://www.alzheimer-europe.org/policy/eu-action) and WHO to facilitate the discovery of disease-modifying treatments through the “*Global Action Plan on the Public Health Response to Dementia 2017–2025*” (World Health Organization, 2017) and its recent implementations, including the recently published Report on Global status (WHO, ISBN: 978-92-4-003324-5).

At present, available treatment options have limited efficacy. Most current therapeutics for AD only transiently improve cognitive symptoms in a minor number of patients (Mauricio et al., 2019). At best, they provide limited cognitive benefit in approximately 40% of people living with dementia, and they have no impact on the underlying disease process or the rate of cognitive decline. While development of symptomatic treatments has slowed, the search for dementia-preventing or dementia-modifying treatments has increased significantly. Very recently, the Food and Drug Administration (FDA) granted accelerated approval for Aduhelm (aducanumab), a human monoclonal antibody that selectively targets aggregated amyloid beta (Aβ). Being a disease modifying therapy, aducanumab holds a great potential for clinical benefit over current symptomatic therapies, however its approval -largely criticized- has been based on the reduction of a surrogate marker (amyloid beta) with questionable data on clinical efficacy (Nisticò and Borg, 2021). Moreover, it is under scrutiny for side effects, including amyloid related imaging abnormalities (ARIA) and brain haemorrhage. In line with this, the European Medicines Agency (EMA) has recently recommended the refusal of the marketing authorisation for Aduhelm (https://www.ema.europa.eu/en/medicines/human/summaries-opinion/aduhelm; EMA 750220/2021).

A plethora of other innovative therapeutic approaches are emerging, with the identification of novel mechanisms as potential drug targets. Indeed, robust and reproducible biological validation of putative new molecular targets is key to successful drug discovery. To date, the success rate for the development of disease-modifying drugs for NDs has been disappointing, like the failure of beta secretase inhibitors or monoclonal antibodies targeting amyloid beta in AD clinical trials (Salloway et al., 2021). This applies also to new drugs directed toward tau including those reducing tau hyper-phosphorylation, tau accumulation or preventing the spread of toxic tau species (Imbimbo et al., 2021).

Here we discuss the main features of neuronal subpopulations and circuits most vulnerable to neurodegenerative insults, and how optimal target engagement is critical for ensuing treatment efficacy. In this frame, we will highlight the importance of the non-invasive intranasal route for brain drug delivery coupled with Focused UltraSound (FUS).

Previous preclinical studies and pilot trials have shown that the intranasal administration of NGF (Tuszynski et al., 2015) and insulin (Craft et al., 2012) to mild cognitive impairment (MCI) and early AD patients was safe and resulted in rapid improvement of cognition, even within 30 min upon nasal sniffing. Noteworthy, and in spite of very promising preclinical data, phase 3 clinical trials investigating NGF and insulin failed, possibly because of the poor target engagement attributed to the implanted device used for continuous delivery of the drugs (Castle et al., 2020; Craft et al., 2020). Comparable promising results in terms of synaptic functions recovery have been obtained by direct BDNF infusion into the enthorinal cortex of animal models of pathology (Nagahara et al., 2013), and this technique has been used also in non-human primates (Nagahara et al., 2018). An open label phase1 clinical trial based on Adeno-Associated Virus (AAV)-Based, Vector-Mediated Delivery of Human Brain Derived Neurotrophic Factor (AAV2-BDNF) in subjects with early AD and MCI has been started by the Tuszynski group last year.

Surely, it is demanding to conceptually reconsider the ND field and take advantage of emerging technical opportunities. Efforts in finding new effective drugs slowing down or halting NDs progression should be coupled with efforts addressed to efficient drug delivery systems. The effectiveness of these methods would be strictly dependent on the administration route and on their intrinsic ability to target organs and tissues in a suitable amount and at the right time. In this perspective, we will describe FUS as a novel non-invasive technical paradigm to allow focused drug delivery to precisely target circuits, with the final aim to reach the optimal drug amount in a specific target area and thus improve the outcome in preclinical and clinical trials.

## Improving Drug Delivery and Target Engagement in NDs: The Promise of FUS

### Drug Delivery Approaches

Different strategies of drug delivery to the brain have been reviewed recently (Wang et al., 2019; Lee and Leong, 2020). The last decade has seen an enormous research effort spent to develop BBB penetration methods using biochemical or physical stimuli, that have also aided in effective preclinical screening of brain targeting therapeutics and external stimulation, among those the application of Magnetic Resonance guided Focused Ultrasound (MRgFUS) is gaining momentum (Leinenga et al., 2016).

Technology, in fact, is in place supporting feasibility of such interventions in humans, and devices using Therapeutic Ultrasound (TU, at high frequency - 620 kHz–1.0 MHz- and intensity) in non-invasive brain surgical ultrasound treatment of NDs were approved by regulatory organisms (FDA and CE mark), and are considered emerging treatment in essential tremor, PD, neuropathic pain and ablation of brain tumours (Leinenga et al., 2016). The same technology, albeit operating at a different frequency (low frequency i.e., 220 kHz, and intensity), is used to allow BBB tailored opening, also known as obicodilation (McDannold et al., 2012). While UT surgery has already reached clinical acceptance status as valid alternative treatment in several CNS (ET, PD) as well as other diseases (uterine fibroma, prostatic cancer, osteoid osteoma, bone tumour), the use of MRgFUS BBB disruption to favour alternative drug administration routes is undergoing a very intense clinical trial activity, summarized in Table 1, for NDs (AD, PD, ALS), and for brain tumours as well.

**TABLE 1:** Current status (August 2021) of Clinical Trials on BBB opening. AD: Alzheimer’s Disease; ALS: Amyotrophic Lateral Sclerosis; PD: Parkinson’s Disease.

	ClinicalTrials.gov IDENTIFIER	Cluster	Objective	Condition	Phase	Patients	Status
1	NCT03119961	1	feasibility, safety of BBB opening in AD patients	AD	I/II	10	completed
2	NCT02986932	1	feasibility, safety of BBB opening with IV administration of US contrast agents in AD patients	AD	I/II	6	completed
3	NCT04118764	1	feasibility, safety of BBB opening with IV administration of US contrast agents in AD patients using US guided neuronavigation guidance	AD	I	6	recruiting
4	NCT03671889	1	feasibility, safety of BBB opening in AD patients	AD	I	20	recruiting
5	NCT04526262	1	feasibility, safety of BBB opening in AD patients	AD	I	6	active
6	NCT03321487	1	feasibility, safety of BBB opening in ALS patients	ALS	I	8	active
7	NCT03626896	1	evaluation of safety and find the tolerated ultrasound dose of transient opening of the blood-brain barrier (BBB)	r-Glioblastoma	I	6	completed
8	NCT03712293	1	evaluation safety and feasibility of BBB disruption along the periphery of tumor resection cavity	Glioblastoma	I	20	recruiting
9	NCT03322813	1	Evaluate the Safety and Feasibility of Temporary Blood-Brain Barrier Disruption (BBBD) in Patients With Suspected Infiltrating Glioma	Glioma	I	15	active
10	NCT03739905	2	feasibility, safety and efficacy of repeated, BBB opening in AD patients	AD	IIa	30	recruiting
11	NCT03608553	2	feasibility, safety and efficacy of repeated, BBB opening in PD patients	PD	I	10	active
12	NCT04370665	3	safety and feasibility of three biweekly delivery of Cerezyme® via BBB opening	PD	I	4	active
13	NCT04528680	3	evaluation of Abraxane® drug crossing of BBB, at increasing doses: dose limitin toxicity and 1-yr survival rate	r-Glioblastoma / Gliosarcoma	I / II	39	recruiting
14	NCT04614493	3	evaluation of Temozolomide drug crossing BBB and efficacy in Glioblastoma patients	Glioblastoma	II	66	recruiting
15	NCT02343991	3	evaluation of Doxorubicin drug crossing BBB and accumulation in brain tumor	brain tumor	I	10	active
16	NCT03616860	3	evaluation of safety of BBB disruption in patients following surgical resection and chemo-radiation with temozolomide (TMZ) protocol	Glioblastoma	I	20	recruiting
17	NCT04998864	3	evaluation of safety and feasibility of BBB disruption in high grade glioma patients under standard of care therapy	Glioma	I	5	recruiting
18	NCT03551249	3	evaluation of safety and feasibility of BBB disruption in high grade glioma patients under standard of care therapy	Glioma	I	20	recruiting
19	NCT03744026	3	evaluate dose limiting toxicity (DLT) of escalating n. of ultrasound beams at constant acoustic pressure and standard escalation (Phase I) safety and efficacy of BBB opening	r-Glioblastoma	IIa	33	active

### FUS Technique

The acoustic pressure delivered locally is the key factor distinguishing the different US-based interventional methodologies: the operative ranges for acoustic pressure and frequencies used in different US therapeutic field applications are schematized in Table 2 (Jun, 2012).

**TABLE 2:** Acoustic pressure (MPa) and Frequency (MHz) dynamic ranges for the focused ultrasound application in current clinical settings for sonothrombolysis, ablative surgery (Essential tremors, PD, and tumour ablation), neurostimulation, Imaging, and obicodilation (BBB opening; bold evidenced).

	Sonothrombolysis	Neuromodulation	Obicodilation (BBB opening)	Imaging	Ablative surgery
Acoustic Pressure (MPa)	0.009–0.300	0.02–0.25	**0.08**–**4.00**	0.1–10.0	1.5–38.0
Frequency (MHz)	0.25–1.15	0.055–1.000	**0.12**–**1.00**	0.85–18.00	0.09–1.05

**FIGURE 1:**
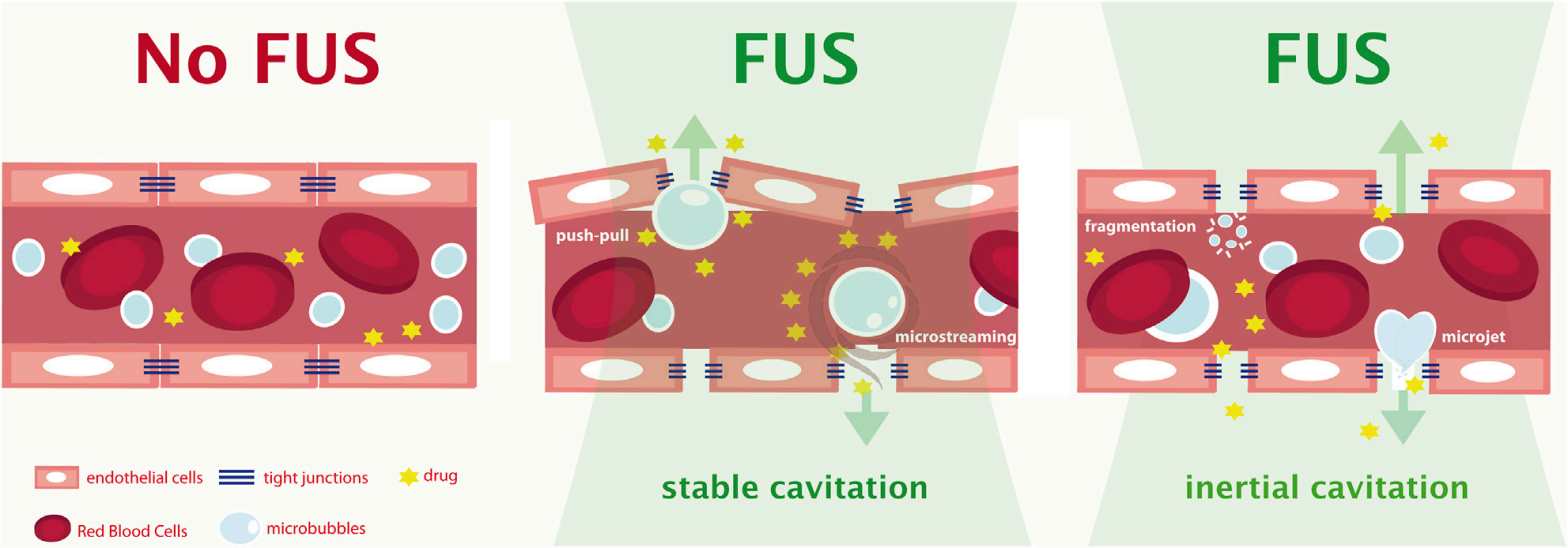
Schematic representation of terminal capillaries targeted by the FUS stream. In the absence of FUS-induced cavitation, an intact BBB of a living animal (No FUS) with flowing red blood cells and peripherally (tail vein) injected microbubbles is depicted. Upon FUS stimulation and depending on the specific combination of acoustic pressure and frequency applied, a controlled stable cavitation with a balanced microbubbles expansion and compression or an inertial cavitation with a wide range of microbubbles diameters may be achieved by the operator at specific brain point locations. Both mechanisms leading to stretching of tight junctions in the endothelial cells of the final capillary allowing a 15′-12 h window of BBB opening and drugs delivery to adjacent brain parenchyma. In case of stable cavitation conditions microbubbles induce the opening of the capillary walls mainly through push-and-pull mechanisms and/or local microstreaming of the blood flow. If inertial cavitation takes place, microbubbles become unstable and collapse emitting high energy microjets or more rarely explode (fragmentation), in both cases facilitating the drug passage through the BBB, however often resulting in local inflammation/oxidative insult.

The use of circulating microbubbles (MB)—i.e., clinically approved contrast agents used for ultrasound imaging - combined with low intensity MRgFUS is an emerging technology which allows to perform a controllable obicodilation (Konofagou et al., 2012; Leinenga et al., 2016; Wu et al., 2020). The mechanism by which BBB disruption takes place is still not entirely elucidated. Capillary diameter varies in the range of 4–10 μm and commonly used MB sizes are in the range 1–5 μm (SonoVue®, Optison®, Definity®, Sonazoid® etc.), thus cyclic repetition of ultrasound bursts inducing MB expansion and contraction, are thought to generate mechanical stress forces disrupting the integrity of BBB tight junctions (Tung et al., 2010; Hosseinkhah and Hynynen, 2012). The focused US beam may induce two types of cavitation phenomena on MB. Stable cavitation resulting from lower/intermediate acoustic pressures promotes a periodic gas decompression/compression within the microbubble inducing their expansion/contraction cycle regularly (Figure 1). In the expansion phase stretching of the vessel may lead to transient opening of the tight-junctions between cells (Dasgupta et al., 2016), while in the contraction phase micro-streams may be produced developing shear stress on the vascular endothelial cells increasing endocytosis (van Bavel, 2007). MB stable cavitation also activates radiation forces pressing and pushing on endothelia and inducing effects leading to increased passive permeability (Dasgupta et al., 2016), Figure 1 *.* The mechanism encompasses a series of complex phenomena including the mechanical disruption of the tight junctions (Sheikov et al., 2004), a reduction of the expression levels of ZO-1, claudin 5 and occludin (Sheikov et al., 2008); an increased number of transcytotic vesicles and increased permeability of cell plasma membrane (Sheikov et al., 2006) and a decrease in drug efflux mechanisms (Cho et al., 2011; Aryal et al., 2017; Choi et al., 2019).

Higher acoustic pressures will lead to inertial cavitation, MB collapse, and consequent microjet formation propagating higher energy shock waves which directed upon endothelia would result in local micro-damages, Figure 1, increasing its permeability at the expense of enormous increased risk of adverse events, i.e. bleeding. Very recently, inertial cavitation finely tuned control is being explored in pre-clinical applications of histotripsy using high-energy very short US pulses also for brain treatment (Lu et al., 2022).

In a series of pioneering studies, the cavitation was reported to produce obicodilation acting on gas particles dissolved in blood. The high energy high frequency focused ultrasound (HIFU) used, however, produced hemorrhage and tissue damage (Vykhodtseva et al., 1995). The introduction of pre-formed MB of the average size 1–5 μm marked an advancement in the field, lowering the amount of energy needed to induce the cavitation allowing to use low energy low frequency Focused Ultrasound (Hynynen et al., 2001; Burgess et al., 2012). Comparing similar amount of BBB disruption obtained applying different US frequencies (0.26–2.04 MHz) to pre-formed MB, resulted in decreased extravasation at the lower frequencies 0.26 MHz, since at higher frequency the threshold of acoustic pressure needed to induce obicodilation is reaching the range needed to engage MB in the inertial cavitation phenomena (Apfel et al., 1991; McDannold et al., 2008).

The dimension of the openings and the recovery time to reseal depend on several factors, the most relevant being the used FUS parameters (McDannold et al., 2008; Baseri et al., 2010 and, 2012; Chopra et al., 2010; Choi et al., 2011), MB type, size and dose (Choi et al., 2010; Tung et al., 2011; McMahon and Hynyen. 2017; Ohta et al., 2020), effective acoustic pressure (Chopra et al., 2010; Samiotaki et al., 2011). Efficiency of BBB opening is strictly related with acoustic pressure and pulse duration, however these parameters are the sensitive ones that relate also with tissue damage in the sonicated region, while MB type, size and dose, together with the pulse repetition frequency (PRF) have been shown to impact the BBB opening without relevant tissue damage after sonication (Shin et al., 2018).

A series of preclinical studies on animal models have shed light on the most relevant physical parameters interfering with the transcranial FUS applications, i.e., the influence of the skull interference has been assessed first in non-human primates (macaques) (McDannold et al., 2008; Arvanitis et al., 2012), and recently in humans (Schwartz et al., 2018; Wang et al., 2018).

As the number of PD and ET patients undergoing to MRgFUS ablative application increased, it was noticed that there were cases were the treatments failed to reach the temperature sufficient to cause a lesion. The analysis of these cases led to the formulation of the concept of Skull Density Ratio (SDR) (Chang et al., 2015). Optimal FUS high intensity energy parameters for patients were since determined taking into account predetermined skull local thickness and SDR ratios, and efficacy of the ablative procedures increased. However, obicodilation is conducted using FUS low intensity energy, and there’s a difference between the attenuation produced when the US wave crosses through the skull if it is a high or a low energy wave. A more recent study based on clinical data available from the obicodilation procedures on human clinical trials has advanced the hypothesis that the trabecular bone ratio showed a significantly greater correlation with dose/delivered energy than that of thickness and the SDR (Kong et al., 2021).

Currently there are three clinical devices approved for brain use in humans: SonoCloud® (CarThera, France), NaviFus® (NaviFUS Corp., Taiwan) and Exablate 4000 (InSightec Ltd., Israel). The first device avoids skull attenuation by positioning the transducer directly in contact on the dural surface, although at the expense of a small craniotomy (Sonabend and Stupp, 2019). The latter two instruments take advantage of the introduction of the phased array transducer technology which has been recently reviewed in depth (Hynynen and Jones, 2016).

Here we will briefly outline the most relevant advantages of the technology: i) it allows transcranial FUS without need of any skull *tomia*, ii) the beam can be “steered” meaning its focal center moved and axis rotated, to different locations in the field space by adjusting the time at which each array element emits the driving signal (i.e., phase shift), iii) wave front aberrations induced by heterogeneous tissue layers crossed can be minimized, iv) using the amplitude and phase of the signals can modify the focus shape or even generate simultaneous multiple foci in different locations. Phased array transducers allow a finer degree of precision positioning the ultrasound focus since, once fixed the mechanical translational positioning of the focal point, can further fine-adjust its positioning with the single elements phase modulation without further mechanical adjustment. Such a possibility is not available on single elements focused transducers.

**FIGURE 2:**
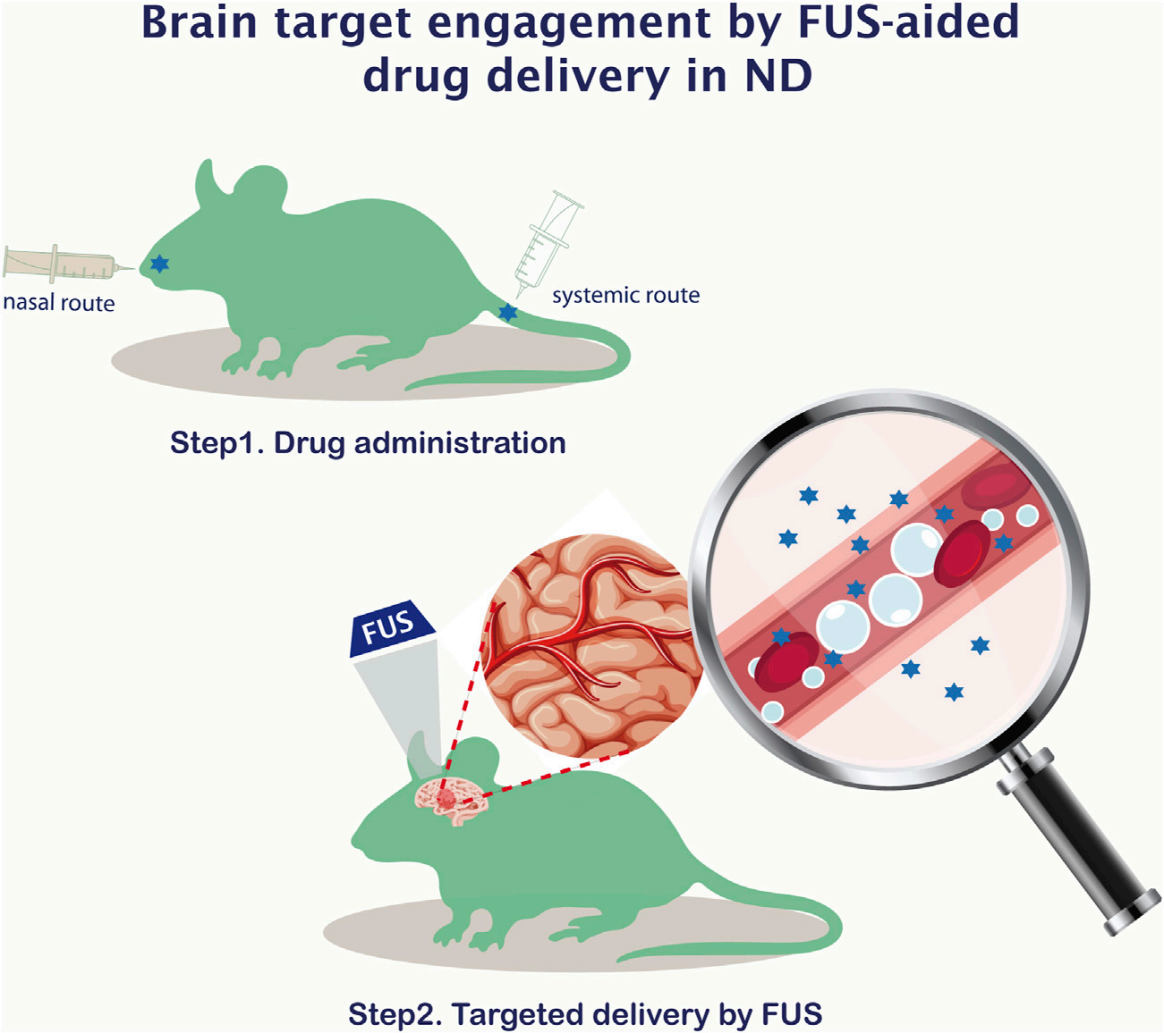
Brain target engagement by FUS-aided delivery of drugs in NDs therapy. A two steps experimental paradigm with the intranasal or systemic administration of the drug of choice, followed by FUS-driven local brain stimulation allowing non-invasive, targeted, and transient opening of the BBB at the region of interest for therapeutic purposes.

**FIGURE 3:**
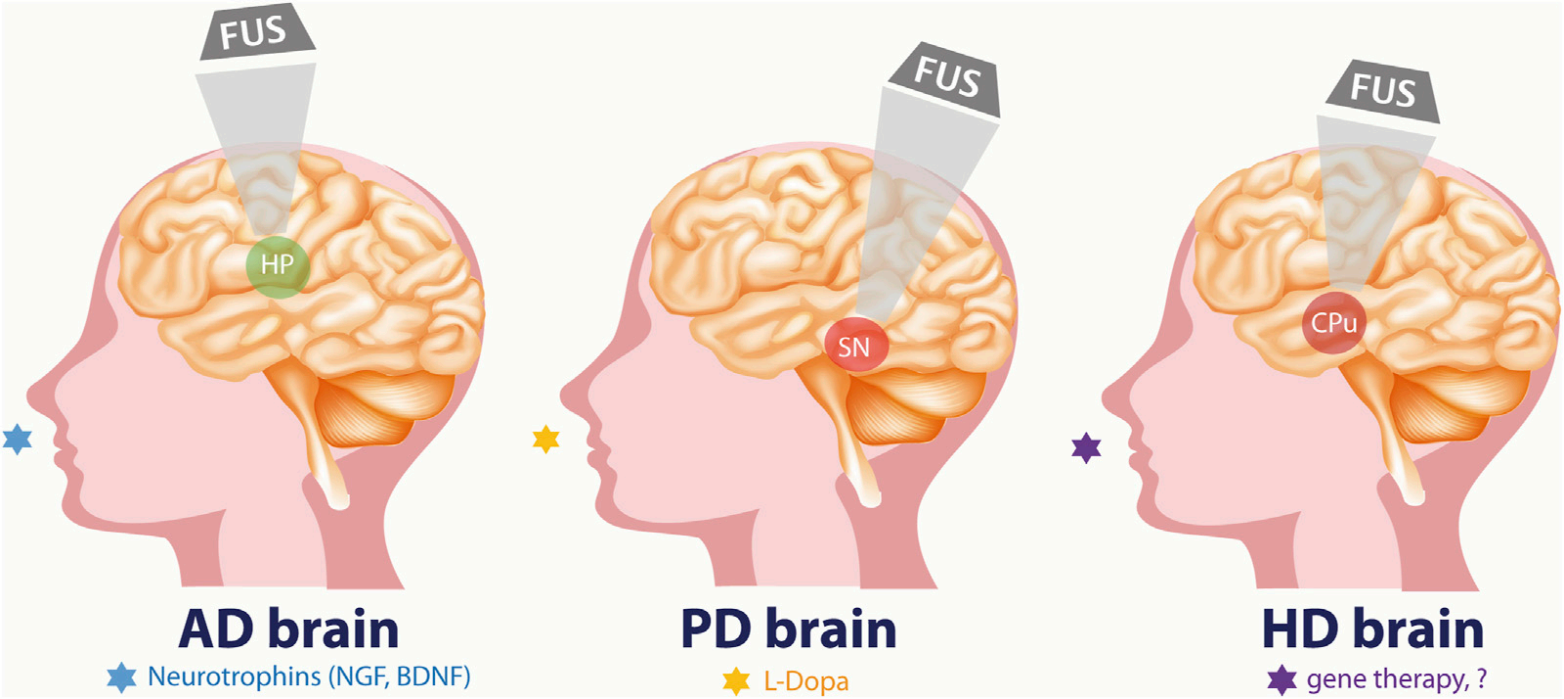
FUS-aided, non-invasive brain delivery of novel or repurposed drugs, as possible therapeutic application in three devastating neurodegenerative diseases of the human central nervous system, namely AD, PD and HD. Specific target regions are identified and selectively reached for FUS-aided drug delivery in neuroprotective/therapeutic approaches: hippocampus (HP) for AD, Substantia nigra (SN) for PD, and Caudate Putamen (CPu) for HD. NGF and BDNF in AD, L-Dopa or dopaminergic drugs in PD, and gene therapy or currently unknown molecules are proposed for intranasal FUS-aided therapy.

Transcranial FUS coupled with the administration of micro-bubbles is proposed as the only non-invasive technique to transiently, locally, and reversibly disrupt the BBB, allowing a temporal and spatial window for molecules to cross to the brain parenchyma both in preclinical animal set-ups (Figure 2) and in patients (Choi et al., 2007; Figure 3).

### FUS in Brain Delivery

A large body of pre-clinical evidence (recently reviewed by Pandit et al., 2020) has been accumulated in the last decade demonstrating that FUS-mediated BBB opening was able to facilitate delivery of a wide variety of therapeutic agents: conventional chemotherapeutics (doxorubicine, methotrexate, temozolomide), mAbs (trastuzumab, bevacizumab), gene therapy vectors, nanoparticles delivering therapeutics for brain tumour treatments, and even whole immune cells (e.g., natural killer cells).

On the side of NDs, the FUS mediated opening of the BBB has been used to explore innovative therapies for AD (Raymond et al., 2008; Jordão et al., 2010; Leinenga and Gotz, 2015, Nisbet et al., 2017; Janowicz et al., 2019), PD (Kinoshita et al., 2006; Lin et al., 2016; Chen et al., 2016; Mead et al., 2017; Yue et al., 2018a,Yue et al., 2018b; Karakatsani et al., 2019), Huntington (Burgess et al., 2012). In august 2021 a survey of the Clinical Trials Database (https://www.clinicaltrials.gov) resulted in a total of 19 studies (Table 1), of which 3 with a completed status (Lipsman et al., 2018; Abrahao et al., 2019; Mainprize et al., 2019), 7 active and nine recruiting. The total number of patients involved so far are 334, for 22 patients the trial has been completed, and for 86 is currently on-going. The above-mentioned completed phase 1 CT has provided the first evidence that this technology is safe in humans under the conditions used. With the increase in the number of clinical studies and recruited patients, the relevance of the secondary effects of the BBB disruption has become a focus of attention. Recent publications have reviewed safety of the transcranial procedure (Pasquinelli et al., 2019) and a first rational overview of the casual effects to brain physiology after BBB disruption (Todd et al., 2020). The former pointed towards a favorable safety profile, while the latter underlined a generalized inflammatory response as the most notable effects, a reduction of both amyloid β plaques and hyper-phosphorylated tau proteins, altered brain transcriptome and proteome profiles, and cerebral blood flow, and finally a transient suppression of neuronal activity. Possible clearance of metabolic waste products through the cerebral spinal fluid (CSF) need further investigations. Notably, the effects reported in the CT are transient with a described time span of few days.

Patients recruited in those studies generally fulfilled the criteria for mild to moderate AD (Lipsman et al., 2018; Meng et al., 2019; Beisteiner and Lozano, 2020), one study targeted early AD patients (Rezai et al., 2020), another patient with PD (Nicodemus et al., 2019) or PD dementia (Gasca-Salas et al., 2021). Although increased BBB permeability was always detected in the targeted regions along with sparse evidence of reduction in Aβ deposition (Park et al., 2021), the clinically meaningful impact of such changes is still questionable (reviewed by Liu et al., 2021) and a sensitive target subpopulation remains elusive.

## Focusing Targets for Neurodegenerative Diseases

In order to be effective, treatments should target early stages of disease. Currently, we lack conceptual frameworks to identify validated biomarkers relevant to disease progression. A deeper knowledge of genetic and environmental selective neuronal vulnerability/resilience is key to discover novel drug targets, appropriate subjects’ selection, and to assess drug-target engagement in clinical trials. Neurodegenerative diseases (NDs) develop over years of progressive metabolic imbalance, synaptic dysfunction and subclinical pathology. Common molecular events include accumulation of a particular misfolded protein, neuronal dysmetabolism, inflammation, oxidative and mitochondrial stress, ultimately leading to neuronal death (Haass and Selkoe, 2007; Frost and Diamond, 2010) and contributing to functional deficits and loss of cognition (Björkqvist et al., 2009).

Although the above-mentioned similarities, NDs differ in their prevalence, age at onset, and clinical characteristics, and particularly in the vulnerability of the neuronal circuits involved (Fu et al., 2018). For instance, high energy demanding and/or high firing neurons, like hippocampal or basal forebrain cholinergic neurons are well-known to be more susceptible to dysmetabolic events and cell stressors. Further, anatomical properties, like the presence of a long extending axon, confer to neurons more vulnerability to chronic stressors, including excess oxidative conditions (Saxena and Caroni, 2011). Activation of extra-synaptic NMDA receptors increasing circuit excitability as a maladaptive response to early injury, possibly boosting neurodegeneration, represents another mechanism of circuit-driven cognitive demise in NDs (Parsons and Raymond, 2014). Also, genetic and epigenetic heterogeneity introduces further interindividual variation. Moreover, environmental insults like brain trauma are potent triggers of neurodegeneration able to turn the initial pathology into a chronic condition (Mendez, 2017; Jamjoom et al., 2021).

### Selectively Vulnerable Brain Circuits

**Alzheimer’s Disease.** AD is the most common neurodegenerative disease, and the most common cause of late-onset dementia (Roussarie et al., 2020). Initial metabolic derangement followed by overt neurodegeneration has been demonstrated to occur in cholinergic circuits innervating frontal cortex and hippocampus, underpinning learning and memory deficits typical of Mild Cognitive Impairment (MCI) and AD (Schliebs and Arendt, 2011; Hampel et al., 2018). Typical AD pathology includes extracellular plaques of amyloid β, and hyper-phosphorylated tau enriched neurofibrillary tangles (Janelidze et al., 2020). Presenilin 1 and 2 mutations characterize early onset familiar AD (Mathews et al., 2000). Amyloid targeting therapy by Adulcanumab has been recently approved by FDA, although controversial for the side effects. No other treatment has been proven to be helpful in halting or delaying the pathology so far.

**Parkinson’s Disease.** PD, the second most common neurodegenerative disease, is a movement disorder, associated to mutations in α-synuclein, LRRK2, parkin, and PINK1, and selectively affecting the substantia nigra dopaminergic (DA) neurons (Surmeier et al., 2017; McGregor and Nelson, 2019). Accordingly, dopaminergic drugs and, in particular, levodopa are current gold standards in PD treatment, although they come with significant side effects. dyskinesia in early onset PD, wearing-off effect, on-off effect, mental symptoms, frozen gait, and last but not least, the irritation and/or other issues at the pump injection site have been reported following chronic levodopa treatment (Vasta et al., 2017).

**Huntington’s Disease.** HD is a fatal genetic disorder affecting muscle coordination and cognition, caused by CAG expansions in the Huntingtin gene and typically involving Huntingtin-enriched inclusion bodies. Striatal medium spiny neurons are selectively vulnerable to HD, resulting in cognitive disabilities early in the disease course, and later progressing to dementia (Rikani et al., 2014; Ruiz-Calvo et al., 2018). No cure is available, and current treatments are mainly symptomatic, including FDA approved tetrabenazine for chorea, antipsychotic drugs and anti-depressant.

**Amyotrophic Lateral Sclerosis** (ALS, or Lou Gehrig’s disease), the most common form of motoneuron disease, is characterized by limb or bulbar initial deficits and leads to progressive paralysis of skeletal muscles.

Spinal alpha-motoneurons, brainstem and upper motor neurons are the specific targets of ALS pathology, which typically presents with deposits enriched in ubiquitin, TDP-43, FUS, and SOD1 (Nijssen et al., 2017). Mutations in SOD1, C9ORF72, TDP-43, FUS, VAPB, and VCP have been described in familial ALS (Taylor et al., 2016; Mejzini et al., 2019). Unfortunately, ALS is an orphan disease, which prognosis is invariably fatal within 3–5 years from diagnosis, with a worldwide incidence of 1.5 individuals per 100.000 yearly worldwide (Xu et al., 2020). ALS manifests as a sporadic disease in 90–95% of ALS affected individuals (Chen et al., 2013). Two stage 3 clinical trials, the first assessing Tofersen called VALOR (Biogen) and based on antisense technologies, and the second with AMX0035 (PHOENIX, Amylix Pharmaceuticals) are currently ongoing (Paganoni et al., 2020).

## Discussion

The most challenging issue of recent therapeutic approaches to CNS pathology is safe, efficient and non- invasive target engagement for disease modifying brain drug delivery.

A number of unsuccessful routes have been attempted so far, including systemic administration, and intracerebral injection of stem cells, or cell encapsulated and growth factors releasing devices. Among the major pitfalls of these approaches are the lack of target selectivity, low entry rates, high amount of drug required, and invasiveness of techniques hindering chronic treatments.

Novel experimental paradigms are needed in order to achieve proof of concept more rapidly than traditional approaches, to reduce the risk of negative outcomes and reduce the overall costs for drug development in NDs. The exploitation of a safe efficient tool to achieve target engagement in specific neuronal circuits is a major challenge in the current neuroscience research.

Focused ultrasound (FUS) is a powerful and precision technique allowing multiple, non-invasive targeted delivery of drugs to the brain. In particular, FUS-aided brain drug delivery through the nasal route has been recently proposed as a paradigmatic model to achieve efficient target engagement in brain pathology (Chen et al., 2016). Clearly, both pro and cons should be weighted for exploitation of the FUS technique for brain drug delivery in humans.

Today three main factors make us believe that FUS mediated disruption of the BBB is more than just another promising tool available to neuro-physicians: 1) a large body of pre-clinical evidence accumulated in the last decade and increasing each year is widening its potential applications in several neurological disorders; 2) the exciting results of the first three completed clinical trials and several others currently being pursued and others in the recruitment phase, with an increasing number of patients that have positively undergone a BBB disruption treatment, including ND patients. At the same time, the current lack of emerging data regarding undesirable side-effects together with the increasing number of studies which are focusing on this issue is definitely pointing towards a realistic wider applicability of the methodology; 3) the technical instrumentation is already available, having gained approval by FDA and CE for specific clinical applications, including medication-refractory essential tremor in 2016, drug-refractory and tremor-dominant PD in 2018, where FUS has indications for non-invasive ablation of the globus pallidus.

Moreover, accumulating evidence pinpoints FUS for both Paclitaxel infusion and ablative surgery in high-grade glioma (Schneider et al., 2020), although some limitations need to be overcome, like skull overheating. Interestingly, regulated BBB disruption by FUS is under investigation for therapy of several NDs, including AD, PD, ALS and BBB opening by FUS has been achieved with success in AD and ALS patients (Elias et al., 2016; Meng et al., 2019; Moosa et al., 2019).

However, more extensive research is warranted regarding possible safety issues. For instance, the prolonged and/or repeated BBB opening might facilitate the brain entry of undesired peripheral immune cells and inflammatory molecules. Also, unexpected mechanical effects, such as focal heating, should be considered in order to reduce the chance of injury along the path. In any case, anatomical and physiological characteristics of each individual should be considered with respect to the capability of BBB opening. Thus exposure parameters should be tailored in order to optimize the amount of acoustic energy delivered while minimizing the potential occurrence of adverse effects.

Noteworthy, despite some minor issues and limitations currently addressed in its clinical use, FUS-aided brain drug delivery is expected to offer significant advantages in clinical settings, like to improve drug pharmacokinetic profile, decrease side-effects, e.g., minimize the risk of haemorrhage and infection compared to more invasive neurosurgical procedures.

In line with its clinical potential, FUS-based brain treatment has been granted by several funding agencies, included NIH, and it is currently under scrutiny for the treatment of ischemic and haemorrhagic stroke, gene therapy and antibody delivery, and neurostimulation, drug-resistant neuropatic pain and trigeminal neuralgia, as recently reviewed (Giammalva et al., 2021).

Nowadays, the application of FUS is gaining particular momentum for brain delivery of current treatments or repurposed drugs. Particularly, FUS application combined with intranasal delivery may be helpful in achieving infusion of neurotrophins, like NGF and BDNF, into specific damaged areas of the brain, of foremost clinical relevance in AD therapy.

NGF or insulin nasal spray have been proposed for human use upon encouraging studies on animal models and humans (Craft et al., 2012; Tuszynski et al., 2015; Manni et al., 2021). However, once at the clinical trial stage, insulin infusion failed to show any effect, supposedly because of the releasing device (Craft et al., 2020). Indeed, NGF based gene therapy has been also attempted in clinical trials, and resulted in failure of cognitive efficacy and/or off-target effects attributed by the authors to the implanted device (Castle et al., 2020).

Noteworthy, levodopa nose-to-brain delivery by nanoparticles (Arisoy et al., 2020) has been interrogated in PD and levodopa inhalation powder (Inbrija, Acorda therapeutics) has been approved by FDA for OFF periods. Thus, FUS-aided intranasal levodopa delivery may be envisaged as a potential FUS application for this devastating brain pathology.

Overall, by allowing targeted delivery of drugs in specific areas of the brain relevant to the different pathologies, the FUS-aided nasal delivery of novel or repositioned drugs may represent a game-changer in treating a wide range of still incurable brain pathological conditions.

## Abbreviations

AD, Alzheimer’s Disease; ALS, Amyotrophic lateral sclerosis; BBB, Blood brain barrier; BDNF, Brain-derived neurotrophic factor; CPu, caudate putamen; CT, computer tomography; ET, essential tremor; EMA, european medicine agency; FDA, Food and Drug Administration; FUS, focused ultrasound; HP, hippocampus; HD, Huntington Disease; MB, microbubbles; ND, neurodegenerative diseases; MCI, Mild Cognitive Impairment; PD, Parkinson’s Disease; NGF, Nerve growth factor; SN, substantia nigra.
